# Effects of Isolated Myofascial Release Therapy in Patients with Chronic Low Back Pain—A Systematic Review

**DOI:** 10.3390/jcm12196143

**Published:** 2023-09-23

**Authors:** Piotr Ożóg, Magdalena Weber-Rajek, Agnieszka Radzimińska

**Affiliations:** Department of Physiotherapy, Collegium Medicum in Bydgoszcz, Nicolaus Copernicus University, 87-100 Torun, Poland; m.weber@cm.umk.pl (M.W.-R.); radziminska@cm.umk.pl (A.R.)

**Keywords:** myofascial release, manual therapy, myofascial system, low back pain

## Abstract

Dysfunctions of the lumbosacral area and related pain syndromes, such as chronic low back pain (CLBP), are among the most common musculoskeletal problems in modern society. The purpose of this study was to evaluate the effectiveness of isolated myofascial release techniques (MFR) in the treatment of CLBP in adults. PubMed, Web of Science, Scopus, and Cochrane Library databases were searched for studies published from 1 January 2013 to 1 March 2023. We included English-language randomized controlled trials evaluating the effect of isolated MFR performed by a specialist on adults with CLBP. Only studies with a comparison group without treatment or with sham MFR were included. A total of 373 studies were detected, of which 6 studies were finally included in this review. There was a total of 397 CLBP patients aged 18–60 in all study groups. The studies evaluated the effects of a series of MFR treatments as well as a single intervention. After applying a series of treatments, a statistically significant reduction in pain intensity, improvement in the range of motion, reduction in the level of functional disability and fear-avoidance beliefs, as well as a decrease in the activity of paraspinal muscles at maximum trunk flexion were demonstrated. A single, 40-min complex intervention involving tissues at various depths significantly reduced the level of pain, improved the range of motion, and reduced the resting activity of paraspinal muscles in the standing position, but did not affect postural stability. The use of a single 5 min MFR technique did not affect pain intensity and sensitivity and functional disability. The findings suggest that the use of a series of isolated MFR improves the condition of patients with CLBP by reducing the intensity of pain, improving functional efficiency, and reducing the activity of the paraspinal muscles in the position of maximum forward bend. The use of a single intervention containing a set of techniques covering superficial and deep tissue also reduces the intensity of pain, improves mobility, and reduces the resting activity of the paraspinal muscles in a standing position. Given the small number of eligible studies with limitations, conclusions should be interpreted with caution and avoid overgeneralizing the benefits of isolated MFR based on limited or mixed evidence.

## 1. Introduction

Dysfunctions of the lumbosacral area and related pain syndromes, such as low back pain (LBP), are among the most common musculoskeletal problems in modern society [1]. Chronic low back pain (CLBP), lasting more than 3 months, is extremely problematic for patients as it significantly limits physical function and quality of life over extended periods of time. Additionally, due to multifactorial etiology, identifying the root cause of the ailment can be challenging [2]. The prevalence of CLBP increases with age, peaking in the 5th and 6th decades of life [3,4]. Although specific causes of pain concern a small percentage of patients, they are more common in young people. On the contrary, up to 90% of LBP is considered to be non-specific low back pain (nLBP) [5]. A prevailing theory suggests that one of the causes of nLBP is myofascial disorder, both local—located in the area of the richly innervated thoracolumbar fascia (TLF)—and distant from the site of pain. Through numerous functional connections, these disorders may affect the lumbosacral spine conditions [6]. Therefore, a therapeutic method that can be used in the treatment of LBP is the myofascial release technique (MFR). MFR is defined as a form of manual therapy focusing on the myofascial system, aimed at mechanically stimulating mechanoreceptors located in the connective tissue, enhancing flexibility, and sliding between layers of soft tissues, thereby reducing muscle activity and pain intensity, and improving patient fitness. From a practical standpoint, MFR involves gradual manual stretching of the patient’s soft tissues (fascia and muscles) in order to remove tension and dysfunction from the myofascial system [7]. 

Research results confirm that even the use of a single, isolated MFR treatment significantly increases the range of motion of the lumbar spine [8], reduces the intensity of pain in individuals with nLBP [9], improves sliding between individual TLF layers [10], enhances blood circulation around paraspinal muscles of the lumbosacral spine [11], and reduces resting activity of the erector spinae and multifidus muscles in this area [12]. 

The aim of this review is to evaluate the effectiveness of MFR as a monotherapy in the treatment of CLBP. The concept of this review arises from our scientific interest in this subject [12,13]. While there are systematic reviews on the evaluation of the effectiveness of MFR in the treatment of LBP, the papers included in them also consider MFR as part of combination therapy or compare its impact with that of other therapeutic methods [14,15].

## 2. Methods

### 2.1. Protocol Registration

The systematic review methods were based on the PRISMA (Preferred Reporting Items for Systematic Review and Meta-Analyses) checklist guidelines [16]. The protocol of this review was regularly validated and registered on PROSPERO (https://www.crd.york.ac.uk/prospero/, assessed on 31 May 2023), with the registration number CRD42023412018. 

### 2.2. Data Sources

The present study is a systematic review of randomized controlled trials (RCTs). We identified studies from the following four databases: PubMed, Web of Science, Scopus, and Cochrane Library, covering the period from 1 January 2013, to 1 March 2023.

### 2.3. Study Selection

Three reviewers (P.O., M.W.R., and A.R.) independently conducted a database search to avoid missing relevant publications. The databases were searched using the following keywords: (1) Myofascial release OR Myofascial release techniques OR Myofascial release therapy OR Myofascial release treatment; and (2) Low back pain OR Chronic low back pain OR Lumbar pain. These two sets of terms were then connected by “AND”. 

Each researcher reviewed only RCTs in English that evaluated the effects of manual therapy based on isolated myofascial release techniques performed by a medical professional on adult patients with chronic low back pain lasting more than 3 months. Only publications with a comparison/control group without treatment or with sham myofascial release therapy were included in the review. 

Studies using myofascial release therapy combined with other therapeutic methods were excluded. Moreover, the self-use of myofascial release by patients with the use of accessories (e.g., rollers or massage balls) was also excluded. The identified reports were critically assessed in terms of quality and relevance. Discrepancies between researchers were discussed until a consensus was reached. 

ZOTERO software version 6.0.22 was used to collect and organize papers, as well as to remove duplicates.

### 2.4. Data Extraction

Two reviewers—P.O. and M.W.R.—independently extracted the data to ensure their reliability and accuracy. Among all eligible studies, the following information was extracted: name of first author, country and year of publication, age of participants, sample size, duration and number of treatments, study design, outcome measures, and main outcomes (findings). Additionally, the description of the study design included details of the duration of follow-up and time points of patient evaluation. Any differences of opinion were resolved through discussion. 

### 2.5. Quality Assessment

To assess the methodological quality of the selected studies, two independent evaluators—P.O. and M.W.R.—used the PEDro scale. The PEDro scale includes the following 11 items: eligibility, randomization, allocation of subjects, similarity at baseline, subject blinding, therapist blinding, assessor blinding, >85% follow-up for at least one key outcome, intention of treatment, statistical comparison between groups, estimated points, and variability measures for at least one key outcome. Each item is rated ‘Yes’ or ‘No’ (1 or 0), depending on whether a criterion is clearly defined in the study. The ratings of items 2 to 11, which determine the internal or statistical validity of the trial, add points to the total PEDro score (range: 0 to 10 points). Higher scores indicate higher methodological quality [17]. Any differences in opinion were resolved through discussion. In case of disagreement, consensus was reached with a third investigator (A.R.).

## 3. Results

### 3.1. Study Selection

**Figure 1:**
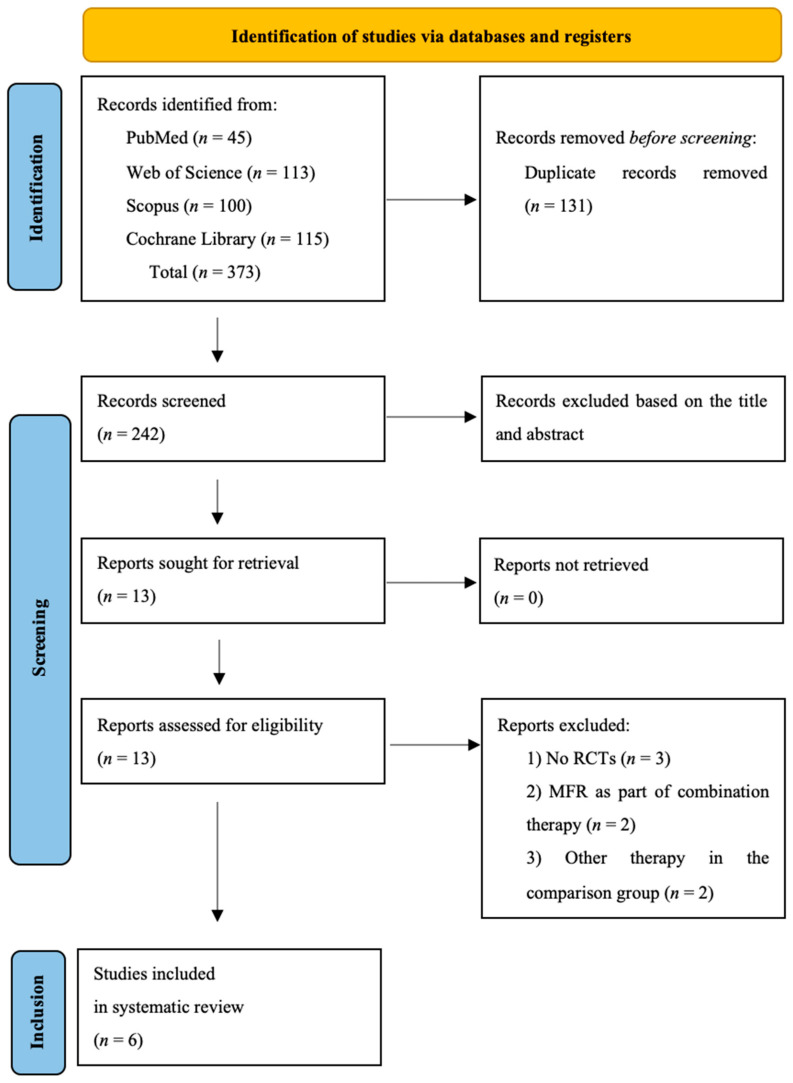
Flow diagram of the study selection process [14].

Using the assumed keywords and considering the range of publication dates, the databases were reviewed, obtaining 373 results. After removing duplicates (*n* = 131), papers were eliminated further by cross-checking stated inclusion and exclusion criteria with study titles and abstracts (*n* = 229), and then, in the next stage, after analyzing full texts (*n* = 8). Ultimately, five papers were qualified for the systematic review. Study identification and screening process are shown in the PRISMA Flow Chart (Figure 1).

### 3.2. Study Characteristics

#### 3.2.1. Overview of Included Studies

The electronic medical databases were searched in April 2023. Table 1 summarizes the characteristics of six RCTs published between January 2013 and March 2023. The total number of participants was 417, with 397 patients diagnosed with chronic low back pain (CLBP) and 20 healthy individuals. Out of these participants, 192 were male and 225 were female, aged 18–60 years old, across all study groups. The studies were conducted in Spain [18,19], Brazil [20,21], and Poland [12,13]. Among all the qualified RCTs, five studies had a parallel design [12,13,18,19,20], and one had a crossover design [21]. 

#### 3.2.2. Intervention Characteristics and Outcome Measures

Table 2 summarizes characteristics of the MFR interventions and outcome measures. In the qualified papers, the authors assessed the effects of both therapies involving a series of MFR treatments with different frequencies and numbers of interventions, as well as the results of a single treatment. Arguisuelas et al. used a two-week therapy with four MFR sessions applied twice a week [18,19], while Sakabe et al. [20] performed three MFR sessions with a frequency of one intervention per week. In both cases, the duration of a single MFR session was 40 min. Paolo et al. and Ożóg et al. assessed the effects of a single intervention, respectively, a complete 40-min MFR session involving multiple manual techniques [12,13] or only one manual technique lasting 5 min (release of the TLF in a seated position with the patient active trunk flexion-extension movement) were applied [21]. Complete MFR sessions included different protocols depending on the authors. Tissues in the area of the lumbosacral spine were manually released at various depths, starting from superficial tissues, such as skin and superficial layers of TLF [12,13,18,19,20,21], iliolumbar ligaments [20], lumbar paravertebral muscles (e.g., erector spinae, ES) [12,13,18,19], and ending with deeper muscles, such as m. quadratus lumborum and m. iliopsoas [12,13,18,19,20]. The authors also used different procedures in the control groups: without any intervention [12,13,20,21], using sham therapy—gently placing the therapist’s hands on the same areas treated in the MFR group, without sliding, and maintaining only contact with the tissues [18,19]—or by asking patients only to perform the same active trunk movements that accompanied manual techniques performed in the experimental group, while renouncing the therapist’s touch [21]. In the paper of Sakabe et al., an additional control group with healthy individuals was also examined [20].

The included RCTs reported different outcomes, which encompassed pain intensity (assessed with SF-MPQ, VAS, NPRS), pain sensitivity (assessed with PPT), range of motion (assessed with Sit and Reach Test, FTF test, measurement of lateral spine inclinations), functional disability (assessed with RMQ, ODI), fear-avoidance beliefs (assessed with FABQ), muscle activity (assessed with sEMG), and postural stability (evaluated through posturography).

All studies measured baseline data before treatment, and in five of them, immediate effects followed the treatment, i.e., after completing a series of MFR treatments [18,19], or in the papers by Paolo et al. and Ożóg et al., immediately after a single intervention [12,13,21]. In a study by Sakabe et al., measurements were taken before treatment, immediately after the first session, then 7 days later at reevaluation, and a month after the end of treatment at follow-up [20]. Follow-up measurements were also performed in one of the studies by Arguisuelas et al. and in both studies by Ożóg et al., respectively, at week 12 [18] or 1 month after the end of MFR therapy [12,13].

### 3.3. Quality Assessment

Based on the PEDro scale, out of six articles that qualified for the review, the highest, ‘excellent’, score was obtained by two papers by Arguisuelas et al. [18,19]. The rest of the papers obtained ‘good’ scores of 6 [20] to 7 points [12,13,21]. Detailed scores for individual criteria are presented in Table 3.

Despite obtaining high scores for methodological quality on the PEDro scale, some papers had shortcomings. Specific issues mentioned include the following cases: two studies did not adequately describe methods for estimating sample size [12,20]; some studies lacked registration or did not publish the research protocol [12,13]; a significant number of the studies were non-blinded [12,13,20,21], which can introduce bias; three of the studies lacked evaluator blinding [12,13,20]; and no studies blinded the therapist performing the MFR procedure.

### 3.4. Synthesis of Results

#### 3.4.1. The Effect of Isolated MFR on Pain

Four RCTs assessed pain intensity and included 171 patients with CLBP. Pain intensity was evaluated using SF-MPQ [18,19], VAS [18,20], as well as NPRS [21]. Additionally, Paulo et al. assessed pain sensitivity using PPT [21]. In the study by Arguisuelas et al., no significant differences in SF-MPQ scores between groups were found immediately after treatment (week 2) [18]. Nevertheless, the results showed a statistically significant decrease in the pain level in the EG, measured by means of the SF-MPQ, compared to the CG at 12-week follow-up (SF-MPQ: mean difference was 7.8, 95% confidence interval [CI]: −14.5 to −1.1, *p* = 0.023, and sensory SF-MPQ subscale mean difference: −6.1; 95% CI [−10.8, −1.5], *p* = 0.011). However, this effect was not observed when assessing pain intensity using the VAS. A statistically significant decrease in the VAS score was found in both groups at week 2 and at week 12. In turn, in the second study by Arguisuelas et al., a significant reduction in pain intensity in the EG (mean difference: 9.1, 95% CI [−16.3, −1.8], *p* ≤ 0.05) was confirmed in the SF-MPQ assessment after the end of treatment (week 2) [19]. The VAS assessment performed by Sakabe et al. confirms a statistically significant reduction of pain intensity (*p* < 0.05) observed immediately after the first MFR intervention (post score), as well as at reevaluation and follow-up (pre-treatment: 3.3 ± 1.9; post-treatment: 1.11 ± 1.4; reevaluation: 1 ± 1.7; follow-up: 0.9 ± 0.9) [20]. The use of a single 5-min MFR technique in the TLF area did not result in any statistically significant changes in the intensity of pain assessed by the NPRS (η2 = 0.32, F = 0.48, *p* = 0.61), as well as pain sensitivity (η2 = 0.73, F = 2.80, *p* = 0.06) [21].

#### 3.4.2. The Effect of Isolated MFR on Range of Motion

In one RCT involving 40 CLBP patients, the range of motion was assessed by measuring the range of lateral inclinations of the spine with a centimeter measure, the Sit and Reach test, and the FTF test [20]. There was a statistically significant improvement (*p* < 0.05) in the Sit and Reach test (pre-treatment: 20.3 ± 7.4 cm, post-treatment: 24.3 ± 7.6 cm, reevaluation: 26.3 ± 8 cm; follow-up: 26.1 ± 7.7 cm) and FTF test (pre-treatment: 13.3 ± 11.33 cm; post-treatment: 8.5 ± 11.5 cm; reevaluation: 4.8 ± 10.5 cm; follow-up: 5.2 ± 10.3 cm).

#### 3.4.3. The Effect of Isolated MFR on Functional Disability

Four RCTs assessed functional disability and included 171 patients with CLBP. Functional disability associated with CLBP was assessed with RMQ [18,19] and ODI scores [20,21]. In the study by Arguisuelas et al., the RMQ score displayed a statistically significant decrease (*p* = 0.03) only at week 12 follow-up in the EG versus CG (MFR-Sham mean difference: −3.7; 95% CI [−7.6, −0.2]). Nevertheless, the authors noted that the extent of the CI does not ensure that the differences observed between groups are clinically important [18]. Differences in the results of RMQ measurements carried out immediately after the end of MFR sessions (week 2) were observed between the studies. While no statistically significant changes in functional disability were observed in the first study [18], in the second study, the measurement carried out post-treatment (week 2) in EG confirmed a statistically significant decrease in functional disability (MFR-Sham mean difference: −5.6, 95% CI [−9.1, −2.1], *p* ≤ 0.05), compared with the CG [19]. The results of the study by Sakabe et al. also confirm a significant reduction in the level of functional disability assessed using the ODI [20]. As a result of the use of a series of three MFR treatments, a significant long-term improvement in ODI index (*p* < 0.05) in reevaluation and follow-up assessments was obtained in the EG (pre-treatment: 15.8 ± 7.3; reevaluation: 9.2 ± 8.6; follow-up: 9 ± 8.5), while in the CG, the level of functional disability remained unchanged. The use of a single 5-minute MFR technique in the TLF area did not result in statistically significant changes in the ODI index (η2 = 0.02, F = 0.02, *p* = 0.97) [21]. 

#### 3.4.4. The Effect of Isolated MFR on Fear-Avoidance Beliefs

One RCT involving 54 patients with CLBP assessed fear-avoidance beliefs using FABQ [18]. The total FABQ score demonstrated a significant decrease in the EG (*p* < 0.05) as compared to sham MFR. Significant differences between groups were observed immediately after the end of the MFR sessions (MFR-Sham mean difference: −14.3; 95% CI [−27.8, −0.8]) and at follow-up (MFR-Sham mean difference: −13.5; 95% CI [−27.6, −0.5]).

#### 3.4.5. The Effect of Isolated MFR on Muscle Activity

Two RCTs involving 149 patients with CLBP assessed muscle activity using sEMG [12,19]. The authors analyzed changes in the resting activity of ES and MF muscles in a standing position [12], as well as changes in the occurrence of the flexion-relaxation phenomenon (FRP), which is characterized by reduced paraspinal muscle activity at maximum trunk flexion (lack of FRP is often associated with LBP). The authors calculated the flexion-relaxation ratio (FRR) by dividing the average EMG activity measured during 85%–100% of the flexion phase by the average EMG activity measured during 45%–60% of the flexion phase [19]. A statistically reliable decrease in the resting activity of ES and MF muscles in a standing position was observed after a single session of MFR therapy. Effects of the treatment were present immediately after receiving the therapy and one month after the intervention. A comparison of the results with those of the CG revealed that the effects were visibly stronger for the MF muscle [12]. Furthermore, there was a statistically significant bilateral reduction in the ES FRR in individuals from the EG who had not shown an FRP at baseline (right M difference = 0.34, 95% CI [0.16, 0.33], *p* ≤ 0.05, and left M difference = 0.45, 95% CI [0.16, 0.73], *p* ≤ 0.05), which indicated an improvement in the FRP response (there was a noticeable reduction in the EMG activity of ES in the full flexion phase) after MFR treatment when compared to the sham group [19]. 

#### 3.4.6. The Effect of Isolated MFR on Postural Stability

One RCT involving 113 patients with CLBP assessed postural stability using a posturography test carried out on a posturographic platform recording and analyzing the center of pressure (COP) movement [13]. The assessment was performed on a stable surface in a free-standing position with eyes open and then closed. The authors analyzed three posturographic parameters, i.e., (1) COP distance [mm] (value of the total COP path length obtained during the study), (2) COP sway area [mm^2^] defined as an elliptic area covering 90% of COP positions), (3) COP sway velocity [mm/s]. It was hypothesized that postural stability would deteriorate immediately after the MFR and improve at follow-up one month after compared with the baseline results. Nevertheless, only 2 out of 12 comparisons of stabilometric parameters showed reliable effects consistent with this hypothesis. Although both comparisons were observed for EG treatment outcomes, there were no reliable differences between the groups.

## 4. Discussion

As presented in the systematic reviews and meta-analyses on the use of MFR in LBP published so far [14,15], several studies used MFR as part of combined therapy, thus complementing other physiotherapeutic effects, such as kinesiotherapy, physiotherapy, and other manual therapy techniques. While such protocols have practical justifications and aim to implement holistic physiotherapy, they also interfere with making objective assessments of individual components of the therapy and determining the most effective interventions. Consequently, the fundamental premise of this innovative systematic review was to include papers examining solely the use of isolated MFR treatment. Our systematic review is limited by a small number of eligible studies, which can be explained by the application of clearly defined inclusion and exclusion criteria. With fewer studies to analyze, it reduces the breadth of evidence available to support the conclusions. The innovative nature of the review in qualifying only studies that used MFR in an isolated form may also be a potential limitation of the work, as it limits the applicability of the findings to broader therapeutic contexts where combined approaches may be used.

Despite obtaining high scores for methodological quality on the PEDro scale, the qualified papers do present certain limitations. It should be emphasized that most of the studies were not blinded [12,13,20,21] and other methodological limitations were observed, such as the lack of adequate presentation of sample size estimation methods [12,20] and the lack of study registration and publication of the research protocol [12,13]. None of the studies blinded the therapist performing the MFR procedure; however, as indicated by Arguisuelas et al. [19], this is due to the nature of the intervention. Interpretation and comparison of results obtained in different studies are challenging due to variations in MFR therapeutic protocols used by authors, including differences in the number and frequency of MFR sessions, as well as the range of manual techniques applied. The observed differences may arise from the lack of standardized guidelines for the implementation of MFR. On the contrary, in studies conducted by Arguisuelas et al. [18,19] and Ożóg et al. [12,13], the researchers applied the same therapy protocol consistently across their subsequent studies. Furthermore, the papers presented in the review adopt varied approaches to intervention in control groups. Sham therapy wherein the therapist’s gestures imitated actual MFR therapy was implemented in the comparison group only in two studies [18,19]. A significant challenge in research involving MFR lies in the standardization of manual therapy, as it is influenced by the quality of the intervention received. Factors such as the skills and experience of the therapist impact their precision in performing the technique and the strength of applied pressure. In only three studies [18,19,21], the authors acknowledged this aspect and discussed the experience of the therapist performing MFR. 

Based on the results obtained, it was observed that 2–3-week sessions of isolated MFR treatment reduced the intensity of pain experienced by individuals with CLBP [18,19,20], enhanced spinal mobility [20], and decreased the level of functional disability [18,19,20], fear-avoidance beliefs [18], as well as paraspinal muscle activity at maximum trunk flexion [19].

It is worth noting that even though authors use the same pain intensity assessments and functional disability questionnaires, and ultimately reach similar conclusions in their papers, they do not always concur regarding the outcomes. Arguisuelas et al. used two different pain intensity questionnaires—SF-MPQ and VAS [18]. The results of only one of the questionnaires (SQ-MPQ) confirmed statistically significant changes in pain intensity. Nevertheless, these were observed only during the follow-up assessment, whereas results obtained immediately after the end of the therapy did not reveal any significant results. On the contrary, in the study conducted by Sakabe et al. [20], statistically significant changes in the level of pain intensity on the VAS scale were observed. Furthermore, it is essential to highlight that the researchers followed another MFR therapy protocol and performed the VAS assessment at a different time. In the second study by Arguisuelas et al. [19], which followed the same MFR protocol as the first one [18], changes in pain intensity level measured with the SF-MPQ were found to be statistically significant immediately after the end of therapy (week 2). However, it is important to note that the first study excluded the assessment of results during this period. Any differences between the studies may potentially arise from variations in sample size—the first study [18] was conducted on a greater number of CLBP patients than the second one [19].

Positive therapeutic effects were observed in not only the following series of MFR but also a single isolated MFR session. After applying a 40-min treatment targeting various superficial tissues and deep muscles in the TLF area, the authors observed both subjective changes, manifested as decreased pain intensity [20], as well as objective improvements in mobility [20] and resting activity of paraspinal muscles in a relaxed standing position [12]. The effects were observed immediately after the intervention and persisted during the follow-up period. Despite that a single 40-min isolated MFR intervention showed no significant effect on postural stability, the researchers highlight that the lack of effect could be attributed to the local nature of the intervention, and they suggest that it might be worthwhile to assess the effects of MFR treatment covering a larger area of the body, including the lower limbs [13]. The 5-min intervention used by Paulo et al., which covered only one technique, did not yield significant changes in terms of pain intensity and functional disability [21]. This suggests that the key aspect contributing to the effectiveness of a single MFR intervention appears to be its sufficiently long duration and the variety of techniques used. It is essential to highlight that the authors evaluated only immediate changes after the delivered intervention while neglecting to assess long-term effects at follow-up.

The findings of this review can serve as the basis for guidelines on the use of isolated MFR interventions in clinical practice. Furthermore, the findings also demonstrate that a series of isolated MFR interventions can improve the condition of patients with CLBP by reducing pain intensity and enhancing their functional fitness. MFR therapy can also be beneficial for patients with demonstrated increased activity of paraspinal muscles during the sEMG exam, especially in conditions involving prolonged standing or forward bending and among patients who experience discomfort during work that requires them to take these positions. Treatment may last from two [18,19] to three weeks [20], with a frequency of one [20] to two MFR interventions [18,19] per week. Nevertheless, if planning a therapy lasting several weeks is not feasible, single MFR interventions should be considered. In such cases, it is crucial to ensure an appropriate duration of the procedure (40 min) and apply techniques targeting soft tissues at various depths, ranging from superficial layers to deeper tissues. When targeting tissues for either MFR treatment sessions or a single MFR intervention, the therapist should be guided by the findings of the functional examination. 

Future studies on isolated MFR should ensure a more rigorous methodology. Subsequent RCTs should strictly adhere to the CONSORT guidelines to minimize bias [22], especially regarding study registration, publishing research protocols, and blinding. We believe that conducting more high-quality RCTs will help validate current findings. It is crucial to establish guidelines for implementing MFR therapy, especially regarding treatment frequency and intervention duration, as this would facilitate comparing future studies. Given the confirmed effectiveness of both a series of MFR sessions and single MFR interventions, it appears justified to conduct a study comparing the effectiveness of these protocols. 

## 5. Conclusions

The findings suggest that a series of isolated MFR treatments have a significant effect on reducing the intensity of pain experienced by people with CLBP, improving the range of motion of the spine, and reducing the degree of functional disability and fear-avoidance beliefs, as well as changes in the activity of paraspinal muscles. Moreover, the use of a single intervention of isolated MFR involving a set of techniques covering superficial and deep tissue layers also reduces pain intensity in the lumbosacral spine, improves range of motion, and reduces resting activity of paraspinal muscles. Although the conclusions drawn from the systematic review seem to support the presented results, given the small number of eligible studies with limitations, they should be interpreted with caution and avoid overgeneralizing the benefits of isolated MFR based on limited or mixed evidence. 

Future research with more rigorous methodologies and consistent protocols with a focus on the safety and technical approach is essential to provide higher-quality evidence to improve the clinical practice of therapists using MFR techniques.

## Figures and Tables

**Table 1:** Study Characteristics.

Authors, Year	Country	Study Design	Mean Age in Years (SD)		Sample Size		Male/Female	
			EG	CG	EG	CG	EG	CG
Arguisuelas et al., 2017 [18]	Spain	Parallel RCT	46.6 (10.3)	46.4 (11.4)	27	27	11/16	10/17
Arguisuelas et al., 2019 [19]	Spain	Parallel RCT	47.2 (9.8)	48.6 (10.1)	18	18	6/12	6/12
Sakabe et al., 2020 [20]	Brazil	Parallel RCT	30.7 (11.2)	CG: 32.1 (13.16) HCG: 27.4 (10.4)	20	20 (CG)/20 (HG)	25/35	
Ożóg et al., 2021 [12]	Poland	Parallel RCT	49.4 (5.9)	48.9 (5.4)	59	54	33/26	26/28
Ożóg et al., 2023 [13]	Poland	Parallel RCT	49.4 (5.9)	48.9 (5.4)	59	54	33/26	26/28
Paulo et al., 2021 [21]	Brazil	Crossover RCT	36		All participants (*n* = 41) underwent three situations in a randomized and balanced order: EG, CG, PG		16/25	

EG—experimental group; CG—control group; HG—healthy group; PG—placebo group.

**Table 2:** Intervention characteristics, outcome measures, and main findings.

Authors, year	Intervention Length, Frequency, and Duration		Outcome Measures	Measurement Time	Main Findings
	EG	CG			
Arguisuelas et al., 2017 [18]	MFR (40 min. each; twice a week; 2 weeks)	Sham MFR	SF-MPQ VAS RMQ FABQ	Pre-treatment, week 2 (post-treatment), week 12 (follow-up)	MFR therapy in the EG resulted in a significant improvement in pain intensity and disability compared to the CG. The authors also concluded that although there were minimal clinically significant differences in pain and disability (within the 95% CI), it remains uncertain whether this improvement is clinically significant
Arguisuelas et al., 2019 [19]	MFR (40 min. each; twice a week; 2 weeks)	Sham MFR	SF-MPQ RMQ sEMG	Pre-treatment, week 2 (post-treatment)	The MFR protocol contributed to normalizing the flexion-relaxation response in individuals who did not show myoelectric silence before the intervention. Additionally, it also showed a significant reduction in pain intensity and disability compared to the CG
Sakabe et al., 2020 [20]	MFR (40 min. each; once a week; 3 weeks)	No intervention	VAS ODI Sit and Reach Test FTF test Measurement of lateral spine inclinations	Pre-treatment, immediately after the 1st MFR session, 7 days after the treatment (reevaluation), 1 month after the treatment (follow-up)	The MFR protocol led to a reduction in pain intensity and lumbar disability degree, along with improved mobility in subjects with CLBP as evaluated through the Sit and Reach and FTF tests. The effects lasted for a month after the end of treatment
Paulo et al., 2021 [21]	MFR (5 min.; single intervention)	CG: no intervention. PG: active trunk movements	NRPS PPT ODI	Pre-treatment, immediately after the MFR session	A single trial of a thoracolumbar MFR was insufficient to reduce pain intensity and disability in subjects with CLBP
Ożóg et al., 2021 [12]	MFR (40 min.; single intervention)	No intervention	sEMG	Pre-treatment, immediately after the MFR session, 1 month after the treatment (follow-up)	A single MFR treatment in the EG led to an immediate decrease in resting activity of the ES and MF muscles in the lumbodorsal spine area. Data collected one month after the treatment confirm the maintenance of the treatment effect in terms of muscular activity of the ES and MF muscles in the lumbosacral spine
Ożóg et al., 2023 [13]	MFR (40 min.; single intervention)	No intervention	Posturography	Pre-treatment, immediately after the MFR session, 1 month after the treatment (follow-up)	A single MFR treatment in the TLF did not aggravate postural stability immediately after the therapy in the EG. Moreover, after one month, postural stability did not improve compared with the results recorded before the treatment. The values of the stabilometric parameters one month after the intervention did not change significantly in the EG compared with the CG

EG—experimental group, CG—control group, MFR—myofascial release, PG—placebo group, SF-MPQ—Short Form McGill Pain Questionnaire, VAS—Visual Analogue Scale, RMQ—Roland-Morris Questionnaire, FABQ—Fear Avoidance Beliefs Questionnaire, sEMG—Surface Electromyography, ODI—Oswestry Disability Index questionnaire, FTF—fingertip-to-floor test, NRPS—Numerical Pain Rating Scale, PPT—pain pressure threshold, ES—erector spinae, MF—multifidus.

**Table 3:** The methodological quality of the included studies (PEDro Scale).

Study/Criteria of the PEDro Scale	Arguisuelas, 2017 [18]	Arguisuelas, 2019 [19]	Sakabe, 2020 [20]	Paulo, 2021 [21]	Ożóg, 2021 [12]	Ożóg, 2023 [13]
Eligibility	+	+	+	+	+	+
Randomization	+	+	+	+	+	+
Allocation of subjects	+	+	-	-	+	+
Similar groups at baseline in terms of the most important prognostic indicators	+	+	+	+	+	+
Blinded subjects	+	+	-	-	-	-
Blinded therapist	-	-	-	-	-	-
Blinded evaluators	+	+	-	+	-	-
Adequate follow-up	+	+	+	+	+	+
Intention of treatment	+	+	+	+	+	+
Comparison between groups	+	+	+	+	+	+
Estimated points and variability	+	+	+	+	+	+
Total score	9	9	6	7	7	7

**Notes:** ‘+’ means the criteria are clearly satisfactory, ‘-’ means the criteria are clearly not satisfactory.

## Data Availability

All data generated or analyzed during this study are included in this published article.

## References

[1] Gandey A. (2009). Low back pain—Normal part of adolescence. Arch. Pediatr. Adolesc. Med..

[2] Treede R.D., Rief W., Barke A., Aziz Q., Bennett M.I., Benoliel R., Cohen M., Evers S., Finnerup N.B., First M.B. (2019). Chronic pain as a symptom or a disease: The IASP Classification of Chronic Pain for the International Classification of Diseases (ICD-11). Pain.

[3] DePalma M.J., Ketchum J.M., Saullo T. (2011). What is the source of Chronic Low Back Pain and does age Play a Role?. Pain Med..

[4] Shmagel A., Foley R., Ibrahim H. (2016). Epidemiology of Chronic Low Back Pain in US Adults: Data from the 2009–2010 National Health and Nutrition Examination Survey. Arthritis Care Res..

[5] Maher C., Underwood M., Buchbinder R. (2017). Non-specific low back pain. Lancet.

[6] Langevin H.M., Fox J.R., Koptiuch C., Badger G.J., Greenan-Naumann A.C., Bouffard N.A., Konofagou E.E., Lee W.N., Triano J.J., Henry S.M. (2011). Reduced thoracolumbar fascia shear strain in human chronic low back pain. BMC Musculoskelet. Disord..

[7] Manheim C.J. (2008). The Myofascial Release Manual.

[8] Marizeiro D.F., Florêncio A.C.L., Nunes A.C.L., Campos N.G., Lima P.O.P. (2018). Immediate effects of diaphragmatic myofascial release on the physical and functional outcomes in sedentary women: A randomized placebo-controlled trial. J. Bodyw. Mov. Ther..

[9] Tozzi P., Bongiorno D., Vitturini C. (2011). Fascial release effects on patients with non-specific cervical or lumbar pain. J. Bodyw. Mov. Ther..

[10] Chen Y.H., Chai H.M., Shau Y.W., Wang C.L., Wang S.F. (2016). Increased sliding of transverse abdominis during contraction after myofascial release in patients with chronic low back pain. Man. Ther..

[11] Shah Y., Arkesteijn M., Thomas D., Whyman J., Passfield L. (2017). The acute effects of integrated myofascial techniques on lumbar paraspinal blood flow compared with kinesio-taping: A pilot study. J. Bodyw. Mov. Ther..

[12] Ożóg P., Weber-Rajek M., Radzimińska A., Goch A. (2021). Analysis of Muscle Activity following the Application of Myofascial Release Techniques for Low-Back Pain—A Randomized-Controlled Trial. J. Clin. Med..

[13] Ożóg P., Weber-Rajek M., Radzimińska A., Goch A. (2023). Analysis of postural stability following the application of myofascial release techniques for low back pain-a randomized-controlled Trial. Int. J. Environ. Res. Public Health.

[14] Chen Z., Wu J., Wang X., Wu J., Ren Z. (2021). The effects of myofascial release technique for patients with low back pain: A systematic review and meta-analysis. Complement. Ther. Med..

[15] Wu Z., Wang Y., Ye X., Chen Z., Zhou R., Ye Z., Huang J., Zhu Y., Chen G., Xu X. (2021). Myofascial release for chronic low back pain: A systematic review and meta-analysis. Front. Med..

[16] Page M.J., McKenzie J.E., Bossuyt P.M., Boutron I., Hoffmann T.C., Mulrow C.D., Shamseer L., Tetzlaff J.M., Akl E.A., Brennan S.E. (2021). The PRISMA 2020 statement: An updated guideline for reporting systematic reviews. BMJ.

[17] De Morton N.A. (2009). The PEDro scale is a valid measure of the methodolo- gical quality of clinical trials: A demographic study. Aust. J. Physiother..

[18] Arguisuelas M.D., Lisón J.F., Sánchez-Zuriaga D., Martínez-Hurtado I., Doménech-Fernández J. (2017). Effects of myofascial release in nonspecific chronic low back pain: A randomized clinical trial. Spine.

[19] Arguisuelas M.D., Lisón J.F., Doménech-Fernández J., Martínez-Hurtado I., Salvador Coloma P., Sánchez-Zuriaga D. (2019). Effects of myofascial release in erector spinae myoelectric activity and lumbar spine kinematics in non-specific chronic low back pain: Randomized controlled trial. Clin. Biomech..

[20] Sakabe F.F., Mazer D.A., Cia J.A., Sakabe D.I., Bortolazzo G.L. (2020). Effects of myofascial techniques on pain, mobility and function in patients with low back pain: A double-blind, controlled and randomized trial. Man. Ther. Posturology Rehabil. J..

[21] Paulo L.R., Lacerda A.C.R., Martins F.L.M., Fernandes J.S.C., Vieira L.S., Guimarães C.Q., Ballesteros S.S.G., Anjos M.T.S.D., Tavares P.A., Fonseca S.F.D. (2021). Can a single trial of a thoracolumbar myofascial release technique reduce pain and disability in chronic low back pain? A randomized balanced crossover study. J. Clin. Med..

[22] Boutron I., Moher D., Altman D., Schulz K., Ravaud P. (2008). Extending the CONSORT statement to randomized trials of nonpharmacologic treatment: Explanation and elaboration. ACP J. Club..

